# Penta‐ALFA‐Tagged Substrates for Self‐Labelling Tags Allow Signal Enhancement in Microscopy

**DOI:** 10.1002/psc.70015

**Published:** 2025-04-13

**Authors:** Souvik Ghosh, Ramona Birke, Ashwin Karthick Natarajan, Johannes Broichhagen

**Affiliations:** ^1^ Leibniz‐Forschungsinstitut Für Molekulare Pharmakologie (FMP) Berlin Germany; ^2^ Max‐Delbrück‐Centrum für Molekulare Medizin (MDC) Berlin Germany

**Keywords:** ALFA‐tag, cell imaging, confocal microscopy, fluorescent labeling, nanobody, self‐labelling proteins (SLPs), signal amplification

## Abstract

Self‐labelling proteins like SNAP‐ and HaloTag have advanced imaging in life sciences by enabling live‐cell labeling with fluorophore‐conjugated substrates. However, the typical one‐fluorophore‐per‐protein system limits signal intensity. To address this, we developed a strategy using the ALFA‐tag system, a 13‐amino acid peptide recognized by a bio‐orthogonal and fluorescently labelled nanobody, for signal amplification. We synthesized a pentavalent ALFA_5_ peptide and used an azidolysine for conjugation with a Cy5‐modified SNAP‐ or HaloTag ligand through strain‐promoted click chemistry. In vitro measurements on SDS‐PAGE showed labelling, and the peptides covalently reacted with their respective tag. HEK293 cells expressing SNAP‐ and HaloTag‐mGluR2 fusion proteins were labeled with ALFA_5_‐Cy5 substrates, and confocal microscopy revealed a significant enhancement in the far‐red signal intensity upon nanobody addition, as quantified by integrated signal density ratios. Comparisons between SNAP‐ and HaloTag substrates showed superior performance for the latter, achieving better signal‐to‐noise and signal‐to‐background ratios, as well as overall signal intensity in plasma membrane‐localized regions. Our results demonstrate the potential of ALFA‐tag‐based systems to amplify SLP fluorescent signals. This strategy combines the photostability of synthetic fluorophores with multivalent labeling, providing a powerful tool for advanced imaging applications including super‐resolution in cells. Its versatility is expandable across diverse protein systems and colors.

## Graphical abstract

A pentaALFA peptide may be reacted with a SNAP‐ or HaloTag and allows for signal enhancement using fluorescently labelled nanobodies. Tagged and peptide/nanobody treated cell surface receptors give generally higher signal intensity using fluorescence microscopy and are amenable for stimulated emission by depletion (STED) super‐resolution imaging.

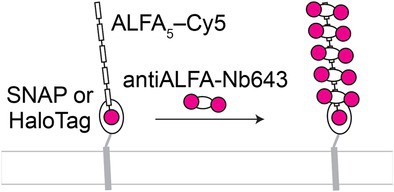

## 1 Introduction

**FIGURE 1:**
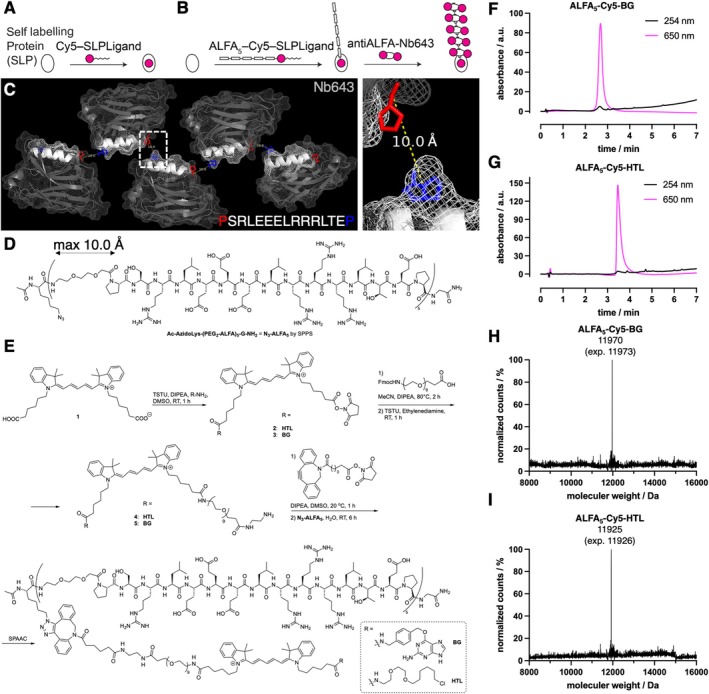
Design and characterization of the penatALFA system. (A) A self‐labelling protein (SLP) is covalently reacted with its specific substrate carrying a dye. (B) Fusing five ALFAtags to the SLP ligand allows the binding of 10 additional fluorophores (2 per Nb). (C) Structure‐guided design shows 10 Å spacing between termini allows PEG_2_ linker usage. (D) Structure of the N3‐ALFA5 peptide, synthesized by SPPS. (E) Chemical synthesis via strain‐promoted alkyne azide click chemistry to obtain ALFA_5_‐Cy5‐HTL and ALFA_5_‐Cy5‐BG. (F) Reverse phase high‐pressure liquid chromatography of ALFA_5_‐Cy5‐BG shows > 97% purity. (G) Mass spectrometry of ALFA_5_‐Cy5‐BG. (H) Reverse phase high‐pressure liquid chromatography of ALFA_5_‐Cy5‐HTL shows > 97% purity. (I) Mass spectrometry of ALFA_5_‐Cy5‐HTL.

Self‐labelling proteins (SLPs) like the SNAP‐ and HaloTag have immensely contributed to the life sciences, in particular in imaging. Appropriate substrate conjugated to fluorophores allow for covalent labelling of protein fusions for subsequent microscopy [1]. Unlike antibodies, these may be used in the live cell setting; however, traditionally, they carry one fluorophore per protein unit (Figure 1A), which gives rise to less obtainable signal intensity. This may hamper the detection of low abundant proteins. This issue has been addressed by for instance using luciferase systems that are able to bioluminesce over prolonged periods of time [2, 3], the use of enzymatic fluorescence signal enrichment by modified ascorbate peroxidase (APEX) [4] or Tyramide Signal Amplification (TSA) [5]. Nonenzymatic signal amplification has been achieved by multimeric fluorescent protein fusions [6, 7], fluorescent signal amplification via cyclic staining of target molecules (FRACTAL) [8], brightness enhancing nanobodies [9], or engineered systems like the SunTag [10] and MoonTag [11], which recruits multiple fluorophores to one site and was recently combined with nanobodies for cell specific signal amplification [12]. DNA‐based system complements the portfolio with for instance immunostaining with signal amplification by exchange reaction (Immuno‐SABER) in the tissue setting [13]. Our aim in this study is to enhance fluorescent signals from self‐labelling proteins using synthetic fluorophores as they are inherently more photostable than fluorescent proteins. We opted to use the ALFA‐tag system [14], which consists of a non‐encoded 13 amino acid long alpha‐helix (H_2_N–PSRLEEELRRRLTEP–OH), flanked by prolines, that is recognized by a bio‐orthogonal nanobody (*K*_*D*_ ~ 25 pM), which each carries two fluorophores according to the manufacturer (Figure 1B, see SI), and has been recently used to enhance fluorescent signals for a CRISPR‐based imaging toolkit [15].

## 2 Materials and Methods

Materials and methods, including all chemical synthesis and characterization, are available in the Supporting Information.

## 3 Results

To firstly design an ALFA_5_ peptide, we aligned the ALFAtag:Nanobody bound X‐ray structures (pdb: 6i2g) [14] in a row, spacing the C‐terminal carbonyl of proline (red) to the N‐terminal nitrogen of proline (blue) to 10 Å (Figure 1C), as these two atoms need to be connected. By doing so, a steric clash‐less arrangement of the protein surface is possible, and gave us confidence to choose a PEG_2_ linker (max. distance ~10 Å). Starting with solid phase peptide synthesis (SPPS), we obtained five ALFA‐tags (ALFA_5_) spaced by such a PEG_2_ linker, which is C‐terminally amidated, and N‐terminally capped by an N‐acetylated unnatural azidolysine, giving a clickable N_3_‐ALFA_5_ (Figure 1D). In parallel, we statistically mono‐conjugated Cyanine5 (Cy5) bis acid to a benzylguanine (BG) or HaloTag ligand (HTL), the substrates for the SNAP‐ and the HaloTag protein (HTP), respectively (Figure 1E). With one free carboxylic acid remaining, we then coupled a PEG_9_ chain with a diethylene amine spacer, before installing a DBCO group. Ultimately, we were able to fuse the ALFA_5_ peptide to the BG/HTL‐Cy5 by strain‐promoted alkyne azide click chemistry in situ, to yield the desired compounds (Figure 1E), ALFA_5_‐Cy5‐BG and ALFA_5_‐Cy5‐HTL, after HPLC purification. We verified purity to be > 97% by LCMS (Figure 1F,G) and characterized the peptides by mass spectrometry (Figure 1H,I).

**FIGURE 2:**
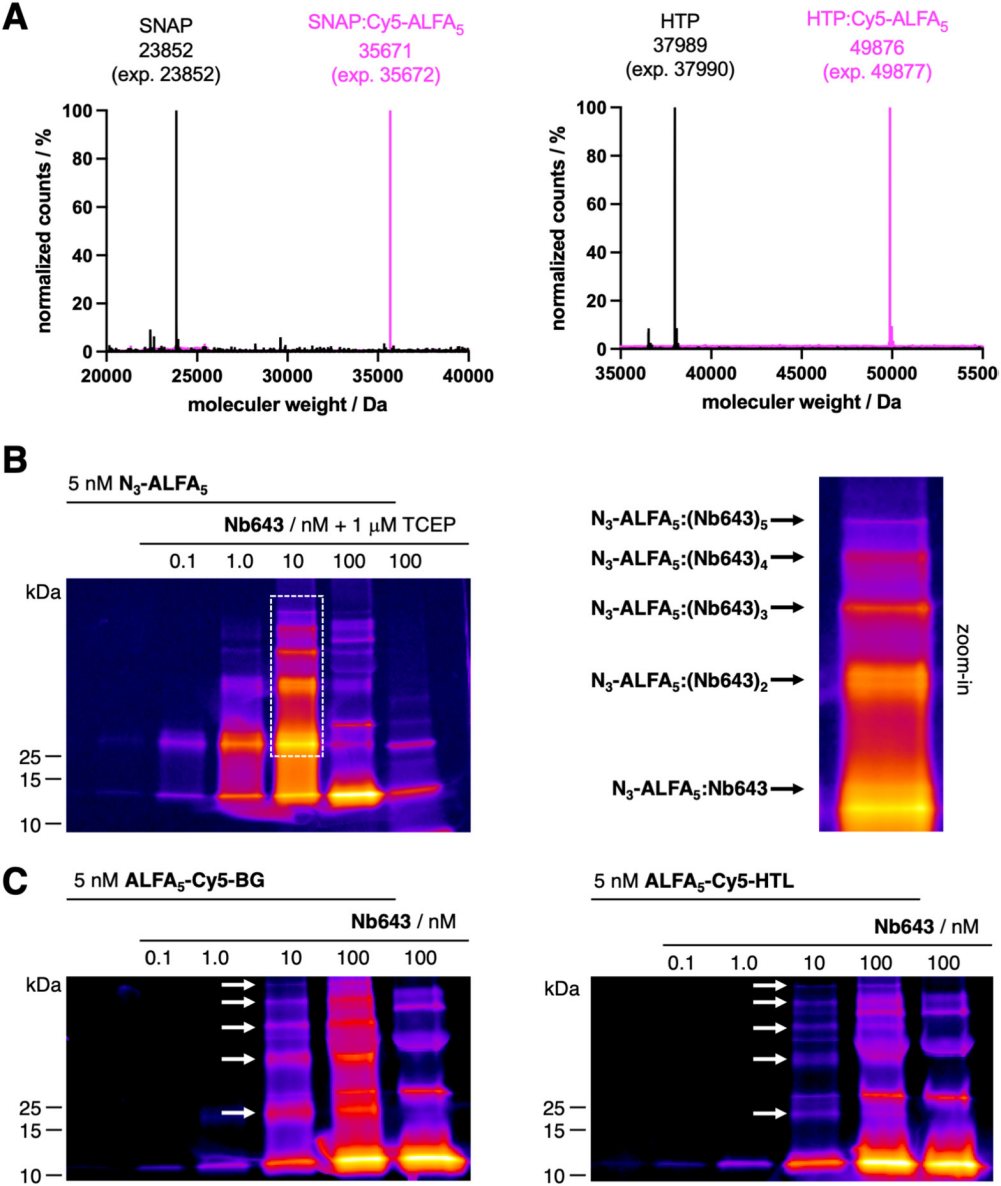
In vitro assessment of the pentaALFA system. (A) Full protein mass spectrometry shows covalent binding of ALFA_5_‐Cy5‐HTL and ALFA_5_‐Cy5‐BG to HTP and SNAP, respectively. (B) Non‐denaturing SDS‐PAGE of N_3_‐ALFA_5_ incubated with varying concentrations of Nb643 shows non‐covalent binding, up to five times as indicated by arrows for the zoom‐in. (C) As for B, but with ALFA_5_‐Cy5‐HTL and ALFA_5_‐Cy5‐BG.

Successful SLP reaction of the respective peptides with a SNAP and HTP was confirmed by prior incubation and successive full protein mass spectrometry (Figure 2A). In order to obtain first insights about Nb binding to the peptides, we incubated 5 nM of N_3_‐ALFA_5_, which does not give any additional fluorescence with respect to our designed SLP‐labelling peptides, with varying concentrations of Nb643 (0.1–100 nM), which was preincubated with TCEP (1 μM) to reduce oligomers and performed a non‐denaturing SDS‐PAGE (Figure 2B). While Nb643 alone still showed some sorts of higher oligomers, we were able to see conjugates at 10 nM Nb643 concentration, which we account for up to five binding events (see zoom‐in). Since TCEP was not able to fully reduce any remaining disulfide bonds, we neglected this step and incubated 5 nM of ALFA_5_‐Cy5‐BG/HTL with varying concentrations of Nb643 in a similar experiment (Figure 2C), which allowed to observe signals that we interpret to five binding events in both cases (black arrows). Therefore, we are able to show that the spacer between the ALFA epitope is sufficient for penta‐Nb binding, yet a 10‐fold signal enhancement deems difficult.

**FIGURE 3:**
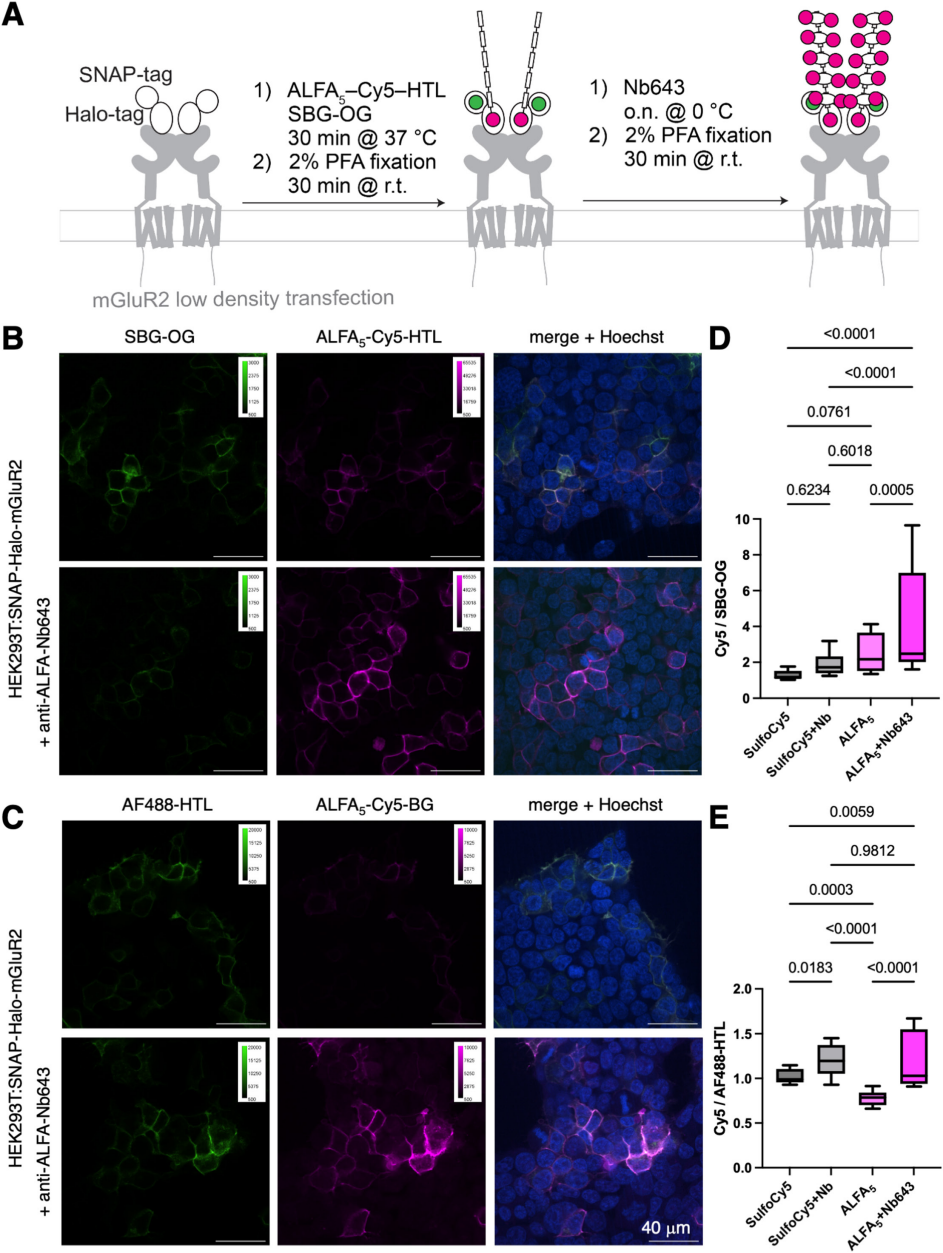
Live cell staining and signal amplification in fixed mammalian cells. (A) Staining protocol of extracellular SNAP‐ and HTP‐tagged mGluR2. (B) SNAP‐HTP‐mGluR2 transfected HEK293 cells labelled with SBG‐OG (for expression control) and ALFA_5_‐Cy5‐HTL. Lower row with addition of antiALFA Nb643. (C) As for B, but stained with AlexaFluo488‐HTL and ALFA_5_‐Cy5‐BG. (D, E) Full image integrated density of far‐red divided by green channel from B and C. SulfoCy5 served as control that does not bind to Nb643. Two biological replicates, 10 images each; min to max box‐and‐whiskers, one‐way ANOVA.

We next wondered if our probes would be amenable to signal enhancement in microscopy. For this reason, we sparsely transfected HEK293 cells (50 ng DNA in an eight‐well ibidi dish) with a SNAP‐HTP‐metabotropic glutamate receptor 2 (SNAP‐HTP‐mGluR2) fusion construct [16]. mGluR2 is a class C G protein‐coupled receptor, involved in neuromodulation and disorders like anxiety [17]. Importantly, the fusion protein contains a SNAP and HTP tag, allowing for labelling one tag with our ALFA‐tag substrate, and the remaining tag with an impermeable green fluorophore (Alexa488‐HTL or SBG‐OG [18]) to normalize for expression levels (Figure 3A). Labeling was performed for 30 min at 37°C (500 nM substrate, 1 μM green fluorophore, 1 μM Hoechst), before we fixed the cells using 2% PFA. Next, we incubated the specimen with 50 nM antiALFA‐Nb643 at 0°C over night, before performing a second fixation step as before. By having one Cy5 fluorophore on the tag, this will allow to determine signal enhancement, while BG/HTL‐SS‐SulfoCy5 (ref [16]) was used a control that should not bind to the nanobody. We then imaged cells treated with SBG‐OG/ALFA_5_‐Cy5‐HTL (Figure 3B) or Alexa488‐HTL/ALFA_5_‐Cy5‐BG (Figure 3C), each ± antiALFA‐Nb643, on a confocal microscope. A signal enhancement with nanobody treatment may already observed by the naked eye (Figure 3B,C; upper versus lower row), yet we quantified the signals by integrating all signal density of the far‐red and green channel, before dividing these values through each other. We observed a significant signal enhancement by adding the nanobody to ALFA_5_‐Cy5‐HTL treated cells (Figure 3D) by a factor of 1.8 (4.5 versus 2.5 for Cy5/OG). Generally, when comparing to SulfoCy5‐HTL treated cells, the obtained values were higher, and although the nanobody has no epitope to bind to in the case for SulfoCy5‐SS‐HTL labelled cells, an enhancement was also observed by a factor of 1.5 (1.9 versus 1.3 for Cy5/OG). For the case of using ALFA_5_‐Cy5‐BG, far‐red over green signal intensities were lower, and enhancement was observed with antibody treatment (1.6‐fold; 1.2 versus 0.8 for Cy5/AF488), however also in the non‐binding SulfoCy5‐SS‐BG control (1.2‐fold; 1.2 versus 1.0) (Figure 3E).

**FIGURE 4:**
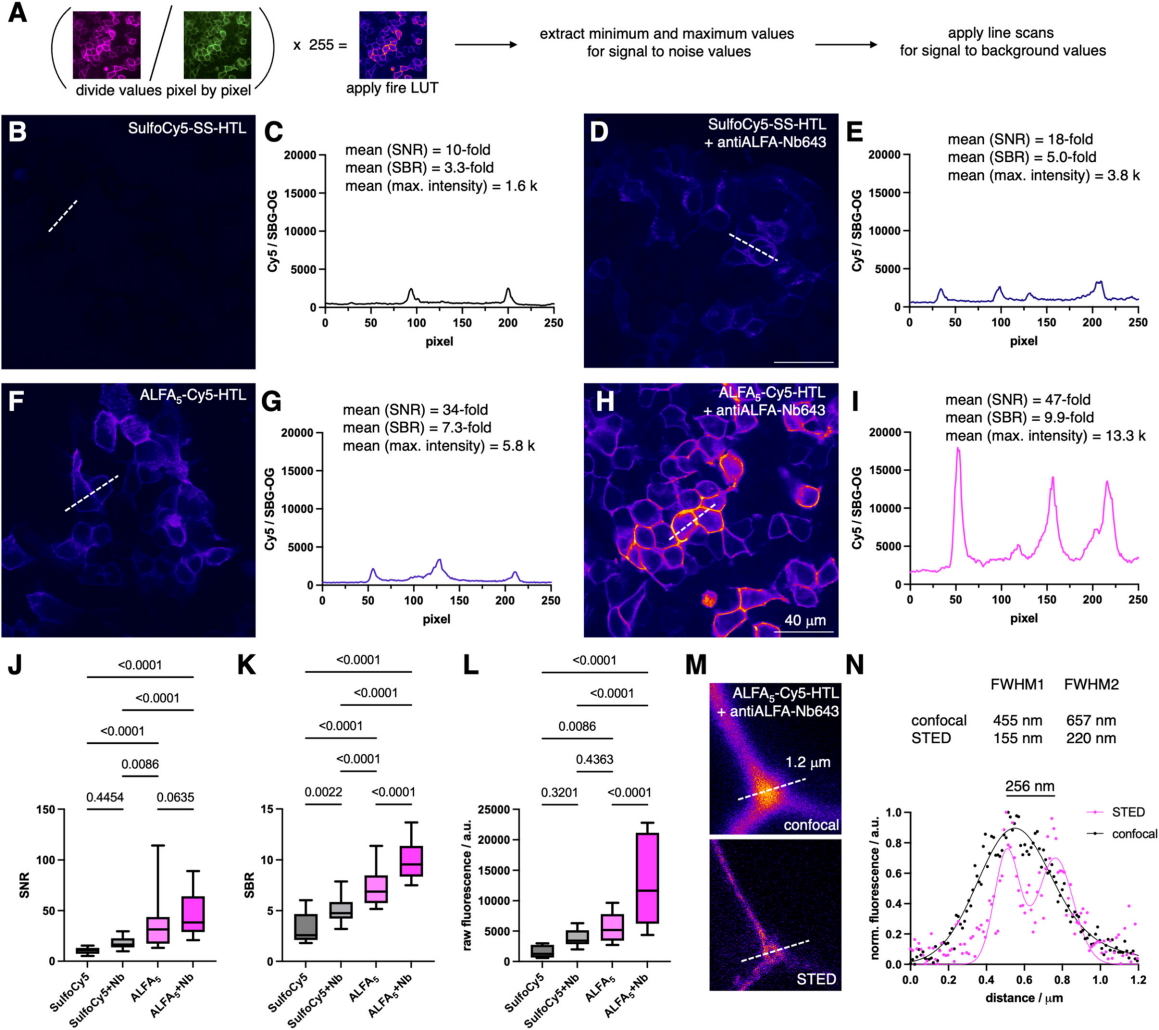
Ratiometric quantification of fluorescent signals and super‐resolution imaging. (A) Protocol for analysis. (B) SNAP‐HTP‐mGluR2 transfected HEK293 cells labelled with AlexaFluor488 (for expression control) and SulfoCy5‐SS‐HTL. (C) Line scan across cells is from B as a representative trace mean values of signal‐to‐noise, signal‐to‐background and maximal measured ratiometric intensity. (D, E) As for B, C but with addition of Nb643. (F) SNAP‐HTP‐mGluR2 transfected HEK293 cells labelled with AlexaFluor488 (for expression control) and ALFA_5_‐Cy5‐HTL. (G) Line scan across cells is from F as a representative trace. (H, I) As for F, G but with addition of Nb643. (J) Quantification of signal‐to‐noise ratio. (K) Quantification of signal‐to‐background ratio. L) Quantification of maximal ratiometric intensity. Two biological replicates, 10 images each; min to max box‐and‐whiskers, one‐way ANOVA. (M) Confocal and STED images of cells from H allow super‐resolution imaging. (N) Line scans from M and sum of two gaussian fitting show resolution beneath the diffraction limit.

While the former experiments were aimed to quantify total signal enhancement, we were interested in the best possible performance. Guided by the fact that the HTL version was more satisfying than the BG substrate, we examined images ± antiALFA‐Nb643 treatment by two different means (Figure 4A), and we anticipate this in particular telling, since we localized the signals to the outer plasmalemma with our strategy. Images were first split into the green and far‐red channel before these were divided through each other pixel by pixel in their intensities to obtain a ratiometric image that was multiplied by a factor of 255 before applying a fire lookup table (LUT). We next measured the minimal and maximal intensity in each image (10–12 images each from two biological replicates to obtain the signal‐to‐noise ratio (SNR). We also performed a line scan through the maximal values over cells from each image to again extract the minimal and maximal values for determining the signal to background ratio (SBR). Exemplary examples are shown for treatment with SulfoCy5‐SS‐HTL, SulfoCy5‐SS‐HTL + Nb643, ALFA_5_‐Cy5‐HTL, and ALFA_5_‐Cy5‐HTL + Nb643 in Figure 4B,D,F,H, respectively, with the inherent line profile shown in Figure 4C,E,G,I. Numbers are given in the latter and are plotted for SNR, SBR, and maximal fluorescent values in Figure 3J,K,L, respectively, with enhancements describing 1.4‐fold (SNR: 47‐fold versus 35‐fold) and 1.4‐fold (SNB: 10.0‐fold versus 7.3‐fold). These experiments recapitulate the trends from Figure 2, yet more precise information can be gleaned about the maximal signal enhancement, and importantly, when comparing the means of the maximal values of ALFA_5_‐Cy5‐HTL versus ALFA_5_‐Cy5‐HTL + Nb643 treatment, generally 2.3‐fold higher fluorescence intensity is gained (mean: 13328 versus 5806). We lastly subjected the ALFA_5_‐Cy5‐HTL ± Nb643 specimen for stimulated emission by depletion (STED) super‐resolution imaging, and while the sample without nanobody remained dim, good confocal signals were obtained when Nb643 was added (Supplementary Figure S1). Acquiring STED images failed for Cy5 through rapid bleaching, yet fine structures were obtained by signals stemming from the nanobody (Figure 4M). A line scan of the membrane contact site showcases the power of super‐resolution imaging, and when fitted to a sum of two gaussians, markedly better full width at half‐maximum distances were obtained for STED (Figure 4N), with a sub‐diffraction resolution of 256 nm. This further highlights the potential of our peptidic probes.

## 4 Discussion

In this study, we present two ligands for the SNAP and HaloTag, each bearing a Cy5 fluorophore and a penta ALFA tag peptide, which can be used to amplify fluorescent signals on tagged proteins by means of a selective nanobody. While more epitopes may be added to the peptide, we concluded for five, since SPPS becomes less efficient the longer the peptide chain, and N_3_‐ALFA_5_ was made in > 80 coupling steps already. Theoretically, this should be able to amplify the Cy5 signal by an order of magnitude, that is, if all five ALFA tags are saturated with a nanobody, which carries two fluorophores according to the vendor. While our ligands successfully labelled SNAP‐ and HaloTags, we conducted a first binding study of ALFA to Nb643 in vitro by co‐incubation, and then running a non‐denaturing SDS‐PAGE. As expected, we observed fluorescent bands of higher molecular weight than the nanobody itself, and when using a two‐fold excess of nanobody, were able to observe five binding events. It should be noted here that (1) the Nb643 is not strictly monomeric, as reported before [19] and confirmed by us using SDS PAGE, and (2) that there is still unbound Nb643 remaining. The latter may be explained by either the semi‐stability of the non‐covalent complex on a gel, and the recently reported ~40% binding efficiency of antiALFA‐Nb to the ALFA tag [20]. Nevertheless, when HEK293T cells were transfected with a low amount of DNA [21], we were able to obtain amplified signals when using our system with the Nb643. Interestingly, the HaloTag outperformed the SNAP‐tag, for which no significant difference was achieved when compared to a non‐Nb643 binding control. We used a green fluorophore for expression control to not engage too much Förster Resonance Energy Transfer (FRET) with the far‐red fluorophores. Generally, we observed higher signals when cells were incubated with Nb643 over night, accounting for non‐specific binding events. Interestingly, ALFA_5_‐Cy5‐HTL was already brighter than SulfoCy5 on HaloTags, possibly induced by the vastly different ligand environment. Lastly, we show that enhanced signal‐to‐background and ‐noise ratios are obtained, when analyzing the images ratiometrically, accounting for a pixel‐by‐pixel expression control. While signals were scaling over a broader range, on average, we were able to boost fluorescent output by 2.3‐fold. Apart from incomplete saturation, another explanation might be that self‐quenching occurs between the fluorophores when assembled onto the peptide, as for instance observed in fluorophore containing dendrimers [22, 23]. Generally, while we have investigated cell surface proteins to label live cells prior to fixation, intracellular targets will be tackled in the future by means of using permeabilizing agents.

## 5 Conclusion

We report on ALFA_5_‐Cy5‐HTL, a HaloTag ligand that can be endowed with antiALFA nanobodies for signal amplification. Given that antiALFA nanobodies are available in different colors, and the use of the ALFAtag has been reported in several systems [24, 25], and the recent endogenous knock‐in of SLPs in vivo [26, 27, 28], we anticipate widespread adoption.

## Author Contributions

SG performed chemical synthesis and characterization. RB, AKN, and JB performed in vitro and cell experiments. JB conceived and supervised the study. JB wrote the manuscript with input from all authors.

## Conflicts of Interest

J.B. receives licensing revenue from Celtarys Research for provision of GLP1R/GIPR chemical probes, unrelated to the present study.

## Supporting information


**Data S1.** Supporting Information.

## Data Availability

The data that support the findings of this study are available from the corresponding author upon reasonable request.
