# Supplementary material for: Penta‐ALFA‐Tagged Substrates for Self‐Labelling Tags Allow Signal Enhancement in Microscopy
Source: J Pept Sci. 2025 Apr 13;31(5):e70015. doi: 10.1002/psc.70015 (PMC11994250; doi:10.1002/psc.70015)
Supplement: Supplementary file 1 — Data S1. Supporting Information. [file PSC-31-e70015-s001.pdf]

Supplemental Information for:

Penta-ALFA-tagged substrates for self-labelling tags allow signal enhancement in microscopy

Souvik Ghosh[1], Ramona Birke[1], Ashwin Karthick Natarajan[2], Johannes Broichhagen[1,*]

---

[1] Leibniz-Forschungsinstitut für Molekulare Pharmakologie (FMP), Robert-Rössle-Str. 10, 13125 Berlin, Germany.
[2] Max-Delbrück-Centrum für Molekulare Medizin (MDC), Robert-Rössle-Str. 10, 13092 Berlin, Germany

To whom correspondence should be addressed:
broichhagen@fmp-berlin.de

Table of Contents

1. General **3**

2. Synthesis **4**

2.1. General Procedure for SPPS *4*

2.2. N_3-ALFA$_5$ *5*

2.3. 2-((1E,3E)-5-((E)-1-(6-((2-(2-((6-Chlorohexyl)oxy)ethoxy)ethyl)amino)-6-oxohexyl)-3,3-dimethylindolin-2-ylidene)penta-1,3-dien-1-yl)-1-(6-((2,5-dioxopyrrolidin-1-yl)oxy)-6-oxohexyl)-3,3-dimethyl-3H-indol-1-ium trifluoro acetate (2) *6*

2.4. 2-((1E,3E)-5-((E)-1-(6-((4-(((2-Amino-9H-purin-6-yl)oxy)methyl)benzyl)amino)-6-oxohexyl)-3,3-dimethylindolin-2-ylidene)penta-1,3-dien-1-yl)-1-(6-((2,5-dioxopyrrolidin-1-yl)oxy)-6-oxohexyl)-3,3-dimethyl-3H-indol-1-ium trifluoro acetate (3) *7*

2.5. 1-(6-((2-(3-((2-Aminoethyl)amino)-3-oxopropoxy)ethyl)amino)-6-oxohexyl)-2-((1E,3E)-5-((E)-1-(6-((2-(2-((6-chlorohexyl)oxy)ethoxy)ethyl)amino)-6-oxohexyl)-3,3-dimethylindolin-2-ylidene)penta-1,3-dien-1-yl)-3,3-dimethyl-3H-indol-1-ium bis trifluoroacetate (4) *8*

2.6. 1-(1-Amino-4,35-dioxo-7,10,13,16,19,22,25,28,31-nonaoxa-3,34-diazatetracontan-40-yl)-2-((1E,3E)-5-((E)-1-(6-((4-(((2-amino-9H-purin-6-yl)oxy)methyl)benzyl)amino)-6-oxohexyl)-3,3-dimethylindolin-2-ylidene)penta-1,3-dien-1-yl)-3,3-dimethyl-3H-indol-1-ium bis trifluoro acetate (5) *9*

2.7. ALFA$_5$-Cy5-HTL *10*

2.8. ALFA$_5$-Cy5-BG *11*

3. Protein expression and purification **12**

4. Protein mass spectroscopy **13**

5. Supplementary Figure **14**

6. Cell culture and imaging **14**

7. Non-denaturating SDS-PAGE **15**

8. Image Calculation and Statistics **15**

9. Excitation and Emission profiles **16**

10. References **16**

1. General

All chemical reagents and anhydrous solvents for synthesis were purchased from commercial suppliers (Sigma-Aldrich, Acros, TCI, IRIS–Biotech, Novabiochem–Merck) and were used without further purification if not stated otherwise. Nb643 was purchased from nano-tag (FluoTag®-X2 anti-ALFA; Cat No: N1502-At643-L), and is reported to carry 2 fluorophores per nanobody (https://nano-tag.com/product/fluotag-x2-anti-alfa/).

NMR spectra were recorded at 300 K in deuterated solvents on a Bruker AVANCE III HD 600 equipped with a CryoProbe or on Bruker AV-III spectrometers using either a cryogenically cooled 5 mm TCI-triple resonance probe equipped with one-axis self-shielded gradients or room temperature 5 mm broadband probe and calibrated to residual solvent peaks ($^{1}H/^{13}C$ in ppm): MeOD-d_4 (3.31/49.00). Multiplicities are abbreviated as follows: s = singlet, d = doublet, t = triplet, q = quartet, p = pentet, h = heptet, br = broad, m = multiplet. Coupling constants *J* are reported in Hz. Spectra are reported based on appearance, not on theoretical multiplicities derived from structural information.

LC-MS was performed on an Agilent 1260 Infinity II LC System equipped with Agilent SB-C18 column (1.8 µm, 2.1 × 50 mm). Buffer A: 0.1% FA in H_2O Buffer B: 0.1% FA acetonitrile. The typical gradient was from 10% B for 0.5 min → gradient to 95% B over 5 min → 95% B for 0.5 min → gradient to 99% B over 1 min with 0.6 mL/min flow. Retention times (t_R) are given in minutes (min). Chromatograms were imported into Graphpad Prism8 and purity was determined by calculating AUC ratios.

Preparative or semi-preparative HPLC was performed on an Agilent 1260 Infinity II LC System equipped with columns as followed: preparative column –Reprospher 100 C18 columns (10 µm: 50 x 30 mm at 20 mL/min flow rate; semi-preparative column – 5 µm: 250 x 10 mm at 4 mL/min flow rate. Eluents A (0.1% TFA in H_2O) and B (0.1% TFA in MeCN) were applied as a linear gradient. Peak detection was performed at maximal absorbance wavelength.

High resolution mass spectrometry was performed on an Agilent Technologies 6230 series accurate mass TOF LC-MS linked to an Agilent Technologies 1290 Infinity Series machine with a Thermo Accucore™ RP-MS column, 2.6 µm pore size, 30 × 2.1 mm, and a 3 min gradient from 5 to 99% aqueous MeCN with 0.1% TFA and MeCN with 0.1% TFA. flow rate: 0.8 mL/min; UV-detection: 220 nm, 254 nm, 300 nm.

2. Synthesis

2.1. General Procedure for SPPS

Peptides were prepared by SPPS on a 50 µM scale using a peptide synthesizer (Automated microwave peptide synthesizer, CEM) and a standard Fmoc/t-Bu protocol. The synthesis was performed on a TentaGel S Ram resin (RappPolymere, Tübingen, Germany). Couplings were achieved by reacting 0.2 M Fmoc-AA-OH with 0.25 M DIC and 0.25 M Oxyma in DMF. A solution of 20% Piperidine in DMF was used to remove the Fmoc protection group. The acetylation is carried out with 3.5 ml DMF, 1 mL acetic anhydride and 0.5 ml DIPA overnight at room temperature. Peptides were deprotected and cleaved from the resin using a mixture of 10 mL TFA, 0.75 g phenol, 0.5 mL water, 0.5 mL methylphenylsulfide, and 0.25 mL 1,2-ethandithiol. After 3 h at room temperature, the cleavage solution was collected, and the crude peptides were precipitated from ice-cold *tert*-butyl-methyl ether. Crude peptides were washed five times with dry diethyl ether. Final RP-HPLC purification and analysis were achieved using a linear solvent gradient (eluent A: 0.05% TFA in water; eluent B: 0.05% TFA in acetonitrile; linear gradient from 5 to 60% B over 30 min at a flow rate of 20 ml·min-1, RT) over a Vydac C18 column (Hesperia, CA, US) and detection takes place at 220 nm.

2.2. N_3-ALFA$_5$

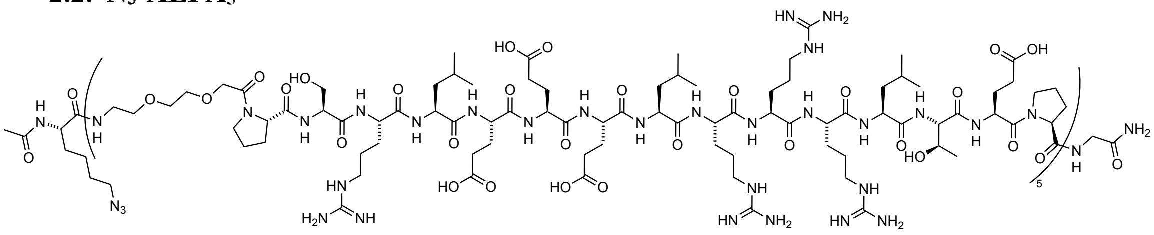

Ac-AzidoLys-(PEG$_2$-ALFA)$_5$-G-NH$_2$ = N_3-ALFA$_5$ by SPPS

<Chromatogram>

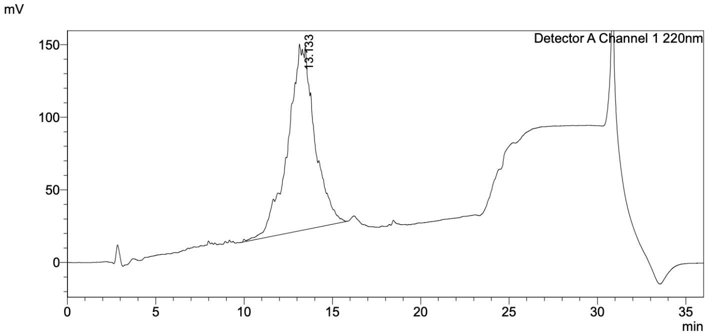

<Peak Table>

Detector A Channel 1 220nm

Peak#	Ret. Time	Area	Height	Conc.	Unit	Mark	Name
1	13.133	13467572	128508	0.000			
Total		13467572	128508				

LRMS (ESI): calc. for $C_{435}H_{761}N_{146}O_{143}$ $[M+13H]^{13+}$: 794.0, found: 794.2; calc. for $C_{435}H_{762}N_{146}O_{143}$ $[M+14H]^{14+}$: 737.3, found: 737.2; calc. for $C_{435}H_{763}N_{146}O_{143}$ $[M+15H]^{15+}$: 688.2, found: 688.5.

2.3. 2-((1*E*,3*E*)-5-((*E*)-1-(6-((2-(2-((6-Chlorohexyl)oxy)ethoxy)ethyl)amino)-6-oxohexyl)-3,3-dimethylindolin-2-ylidene)penta-1,3-dien-1-yl)-1-(6-((2,5-dioxopyrrolidin-1-yl)oxy)-6-oxohexyl)-3,3-dimethyl-3*H*-indol-1-ium trifluoro acetate (2)

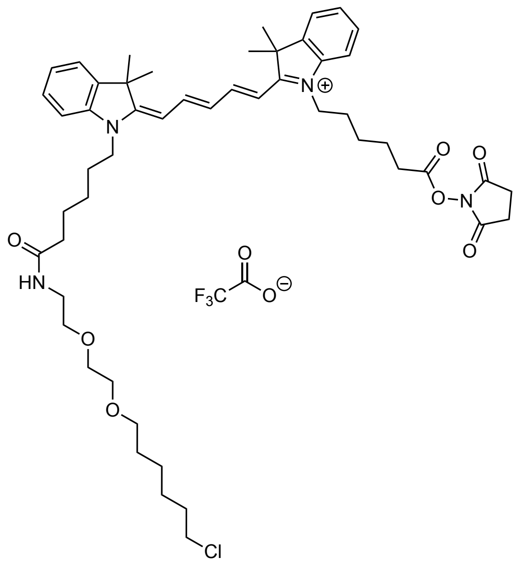

TSTU (45.4 mg, 151 µmol, 2.2 equiv.) and DIPEA (35.8 µL, 205.8 µmol, 3.0 equiv.) were added to compound Cy5-bis acid (40.0 mg, 68.6 µmol, 1.0 equiv.) in DMSO (2 mL) and stirred it for 30 mins before HTL-NH_2 (10.6 µL, 48.2 µmol, 0.70 equiv.) was added. The reaction mixture was stirred for another 1 h before quenched with 2 mL of water, 40 µL of acetic acid and subjected to RP-HPLC purification. The product containing fractions were pooled and the desired product was obtained after lyophilization (17 mg, 17.0 µmol, 25%) as a blue powder.

HRMS (ESI): calc. for $C_{51}H_{71}ClN_4O_7$ $[M]^+$: 886.5000, found: 886.5030.

LCMS traces:

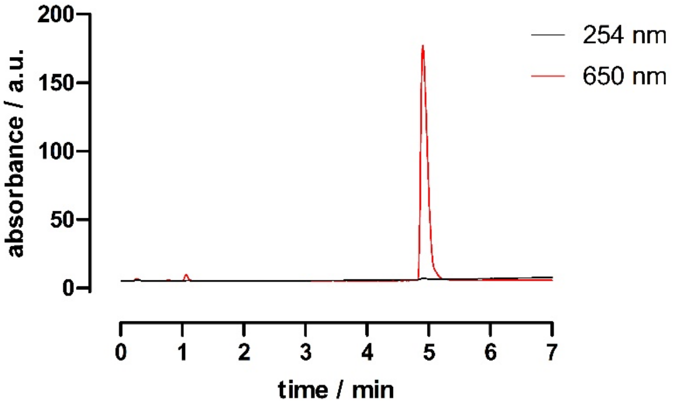

2.4. 2-((1*E*,3*E*)-5-((*E*)-1-(6-((4-(((2-Amino-9*H*-purin-6-yl)oxy)methyl)benzyl)amino)-6-oxohexyl)-3,3-dimethylindolin-2-ylidene)penta-1,3-dien-1-yl)-1-(6-((2,5-dioxopyrrolidin-1-yl)oxy)-6-oxohexyl)-3,3-dimethyl-3*H*-indol-1-ium trifluoro acetate (3)

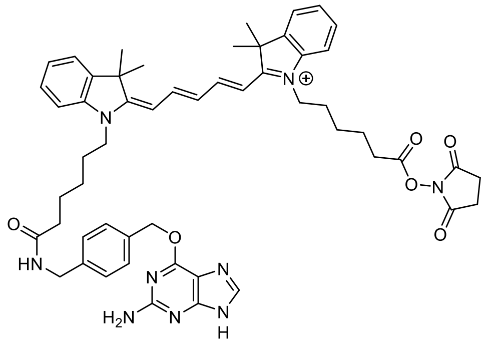

TSTU (22.7 mg, 75.5 µmol, 2.2 equiv.) and DIPEA (17.9 µL, 103 µmol, 3 equiv.) were added to the compound Cy5-bis acid (20.0 mg, 34.3 µmol, 1 equiv.) in DMSO (2 mL) and stirred it for 30 mins before BG-NH_2 (5.56 mg, 20.6 µmol, 0.6 equiv.) was added. The reaction mixture was stirred for another 1 h before quenched with 2 mL of water, 40 µL of acetic acid and subjected to RP-HPLC purification. The product containing fractions were pooled and the desired product was obtained after lyophilization (8.6 mg, 8.2 µmol, 24%) as a blue powder.

LRMS (ESI): calc. for $C_{54}H_{63}N_9O_6$ $[M+H]^{2+}$: 466.7445, found: 466.7471.

LCMS traces:

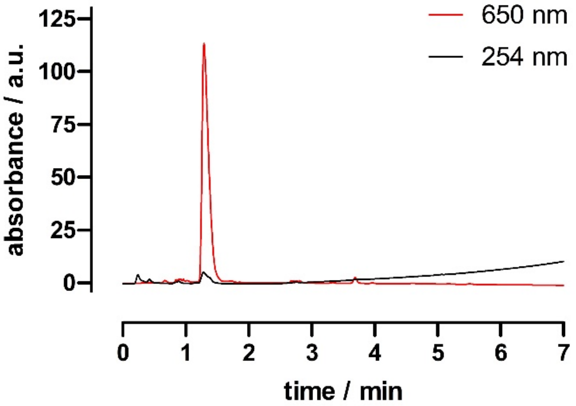

2.5. 1-(6-((2-(3-((2-Aminoethyl)amino)-3-oxopropoxy)ethyl)amino)-6-oxohexyl)-2-((1*E*,3*E*)-5-((*E*)-1-(6-((2-(2-((6-chlorohexyl)oxy)ethoxy)ethyl)amino)-6-oxohexyl)-3,3-dimethylindolin-2-ylidene)penta-1,3-dien-1-yl)-3,3-dimethyl-3*H*-indol-1-ium bis trifluoroacetate (4)

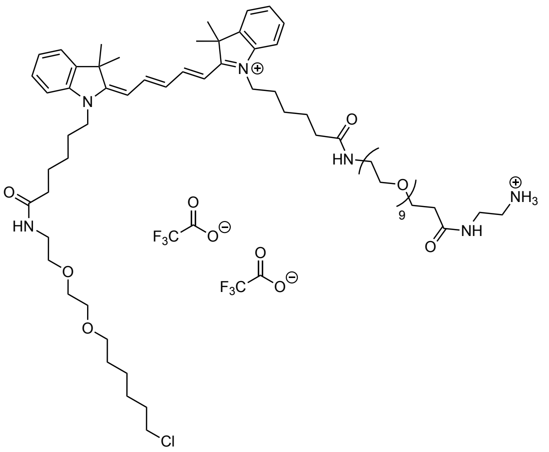

Compound **2** (15.0 mg, 16.9 µmol, 1 equiv.), Fmoc-PEG$_9$-COOH (23.9 mg, 33.8 µmol, 2 equiv.) and DIPEA (17.7 µL, 101 µmol, 6 equiv.) were dissolved in acetonitrile (2 mL) and stirred at 80 °C for 2 h. The reaction mixture was cooled down to room temperature. Then, TSTU (10.2 mg, 16.9 µmol, 2 equiv.) was added to the reaction mixture and stirred at 20 °C for 30 min before ethylene diamine (1.13 µL, 16.9 µmol, 1 equiv.) was added. The reaction mixture was stirred further for another 1 h before quenched with 2 mL of water, 40 µL of acetic acid and subjected to RP-HPLC purification. The product containing fractions were pooled and the desired product was obtained after lyophilization (7.6 mg, 4.97 µmol, 29%) as a blue powder.

HRMS (ESI): calc. for $C_{70}H_{115}ClN_6O_{14}$ $[M+H]^{2+}$: 649.4075, found: 649.4038.

LCMS traces:

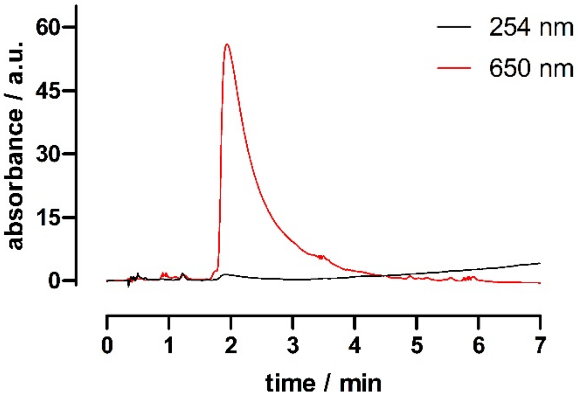

2.6. 1-(1-Amino-4,35-dioxo-7,10,13,16,19,22,25,28,31-nonaoxa-3,34-diazatetracontan-40-yl)-2-((1*E*,3*E*)-5-((*E*)-1-(6-((4-(((2-amino-9*H*-purin-6-yl)oxy)methyl)benzyl)amino)-6-oxohexyl)-3,3-dimethylindolin-2-ylidene)penta-1,3-dien-1-yl)-3,3-dimethyl-3*H*-indol-1-ium bis trifluoro acetate (5)

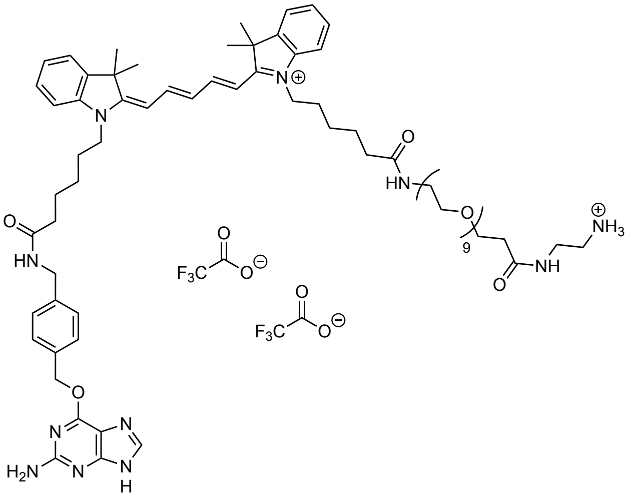

Compound **3** (7.0 mg, 7.50 µmol, 1 equiv.), Fmoc-PEG$_9$-COOH (10.6 mg, 15.0 µmol, 2 equiv.) and DIPEA (7.84 µL, 45.0 µmol, 6 equiv.) were dissolved in acetonitrile (1 mL) and stirred at 80 °C for 2 h. The reaction mixture was cooled down to room temperature. Then, TSTU (4.52 mg, 15.0 µmol, 2 equiv.) was added to the reaction mixture and stirred at 20 °C for 30 min before ethyl diamine (0.50 µL, 7.50 µmol, 1 equiv.) (from stock solution of 2 µL in 10 µL of DMSO) was added. The reaction mixture was stirred further for another 1 h before quenched with 1 mL of water, 20 µL of acetic acid and subjected to RP-HPLC purification. The product containing fractions were pooled and the desired product was obtained after lyophilization (2.80 mg, 1.8 µmol, 24%) as a blue powder.

HRMS (ESI): calc. for $C_{73}H_{108}N_{11}O_{13}$ $[M+2H]^{3+}$: 448.9371, found: 448.9323.

LCMS traces:

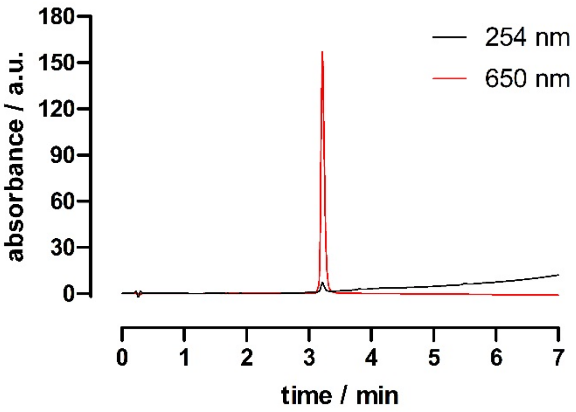

2.7. ALFA$_5$-Cy5-HTL

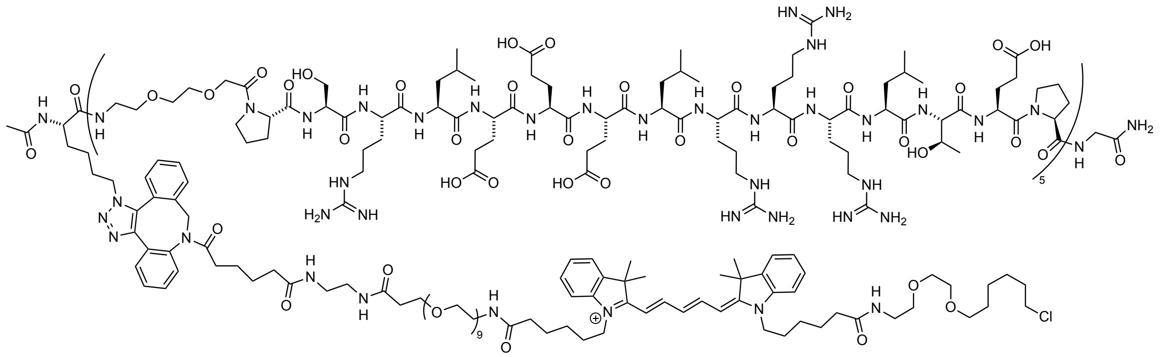

DBCO-NHS ester (0.66 mg, 1.54 µmol, 1 equiv.) in DMSO (10 µL) (from the fresh stock solution of 1.32 mg in 20 µL of DMSO) was added to compound **4** (2.0 mg, 1.54 µmol, 1 equiv.), DIPEA (1.07 µL, 6.16 µmol, 4 equiv.) in DMSO (50 µL) and stirred at 20 °C for 30 min. Then, 15 µL of reaction mixture was taken off and added to the N_3-(PEG$_2$-ALFA)$_5$ (7.78 mg, 0.58 µmol, 1.5 equiv.) in distilled H_2O (50 µL). The reaction mixture was stirred further for 6 h before 65 µL of acetonitrile, 1 µL of acetic acid were added and subjected to RP-HPLC purification. The product containing fractions were pooled and the desired product was obtained after lyophilization (1.98 mg, 165 nmol, 19%) as a blue powder.

HRMS (ESI): calc. for $C_{526}H_{879}ClN_{153}O_{159}$ $[M]^+$: 11926.2, found: 11924.8.

LCMS traces:

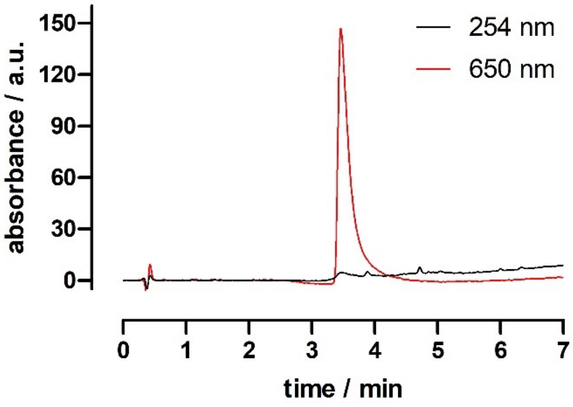

2.8. ALFA$_5$-Cy5-BG

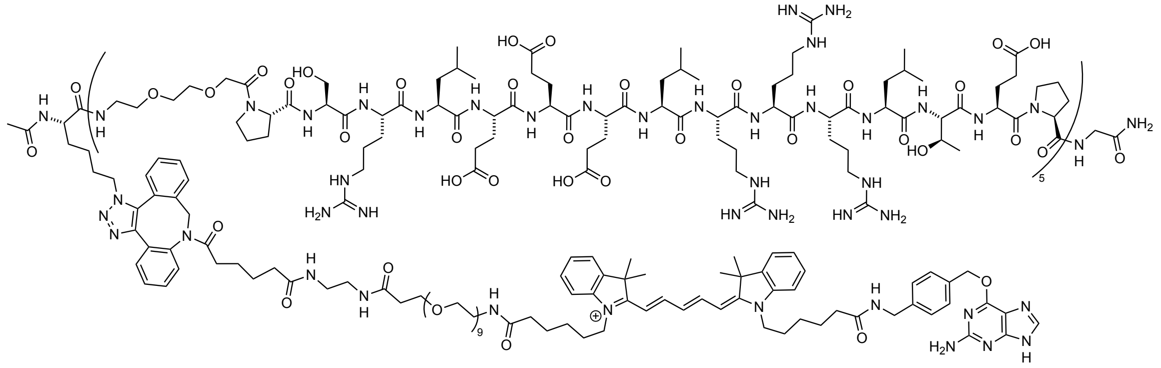

DBCO-NHS ester (0.32 mg, 0.74 μmol, 1 equiv.) in DMSO (10 μL) (from the fresh stock solution of 1.00 mg in 30 μL of DMSO) was added to compound **5** (1.00 mg, 0.74 μmol, 1 equiv.), DIPEA (0.52 μL, 2.96 μmol, 4 equiv.) in DMSO (30 μL) and stirred at 20 °C for 30 min. Then, 20 μL of reaction mixture was taken off and added to the N_3-(PEG_2-ALFA)$_5$ (4.67 mg, 0.45 μmol, 1.5 equiv.) in distilled water (50 μL). The reaction mixture was stirred further for 6 h before 70 μL of acetonitrile, 1 μL of acetic acid were added and subjected to RP-HPLC purification. The product containing fractions were pooled and the desired product was obtained after lyophilization (1.68 mg, 140 nmol, 20%) as a blue powder.

HRMS (ESI): calc. for $C_{529}H_{871}N_{158}O_{158}$ $[M]^+$: 11972.7, found: 11970.2.

LCMS traces:

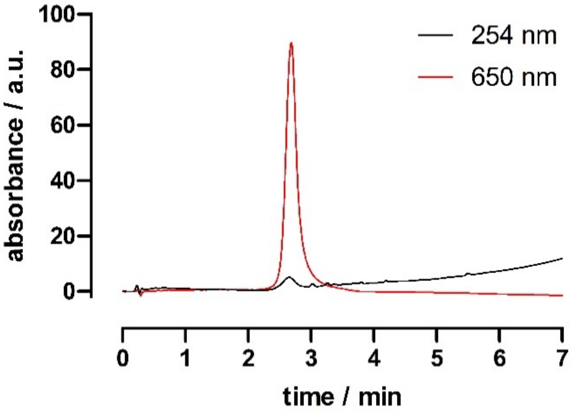

3. Protein expression and purification

$SNAP_f$ and HTP were expressed and purified as described previously.[1] Briefly, proteins were expressed in *E. coli* strain BL21 (DE3). LB media contained ampicillin (100 µg/mL) for protein expression. A culture was grown at 37 °C until an OD_{600} of 0.6 was reached at which point cells were induced with IPTG (1 mM). Protein constructs were expressed overnight at 16 °C. Cells were harvested by centrifugation and sonicated to produce cell lysates. The lysate was cleared by centrifugation and purified by Ni-NTA resin (Thermofisher) according to the manufacturer's protocols. Purified protein samples were aliquoted in PBS, flash frozen and stored at –80 °C.

$SNAP_f$ sequence:

```
MASWSHPQFEKGADDDDKVPHMDKDCEMKRTTLDSPLGKLELSGCEQGLHEIIFLGKGT
SAADAVEVPAPAAVLGGPEPLMQATAWLNAYFHQPEAIEEFPVPALHHPVFQQESFTRQ
VLWKLLKVVKFGEVISYSHLAALAGNPAATAAVKTALSGNPVPILIPCHRVVQGDLDVG
GYEGGLAVKEWLLAHEGHRLGKPGLGAPGFSSISAHHHHHHHHHH
```

Strep-Tag II, Enterokinase-site, $SNAP_f$, His-Tag

HTP sequence:

```
MASWSHPQFEKGADDDDKVPHGSEIGTGFPFDPHYVEVLGERMHYVDVGPRDGTPVLFL
HGNPTSSYVWRNIIPHVAPTHRCIAPDLIGMGKSDKPDLGYFFDDHVRFMDAFIEALGL
EEVVLVIHDWGSALGFHWAKRNPERVKGIAFMEFIRPIPTWDEWPEFARETFQAFRTTD
VGRKLIIDQNVFIEGTLPMGVVRPLTEVEMDHYREPFLNPVDREPLWRFPNELPIAGEP
ANIVALVEEYMDWLHQSPVPKLLFWGTPGVLIPPAEAARLAKSLPNCKAVDIGPGLNLL
QEDNPDLIGSEIARWLSTLEISGAPGFSSISAHHHHHHHHHH
```

Strep-Tag II, Enterokinase-site, HTP, His-Tag

4. Protein mass spectroscopy

Labelling substrates were dissolved in DMSO to a concentration of 1 mM and diluted in PBS (pH = 7.4) to obtain a centration of 20 μM. Protein were diluted in PBS to a concentration of 10 μM. 20 μL of each protein and labelling agent were combined in a mass spec vial and allowed to incubate at room temperature for 1 h, before full protein mass was acquired. For non-labelling control, 20 μL of PBS was mixed with 20 μL of each protein.

Conditions	Calculated	Found
HTP	37989	37989
$ALFA_5$-Cy5-HTL	11926	11924
HTP:Cy5-$ALFA_5$	49878	49876
SNAP	23853	23852
$ALFA_5$-Cy5-BG	11972	11970
SNAP:Cy5-$ALFA_5$	35674	35671

5. Supplementary Figure

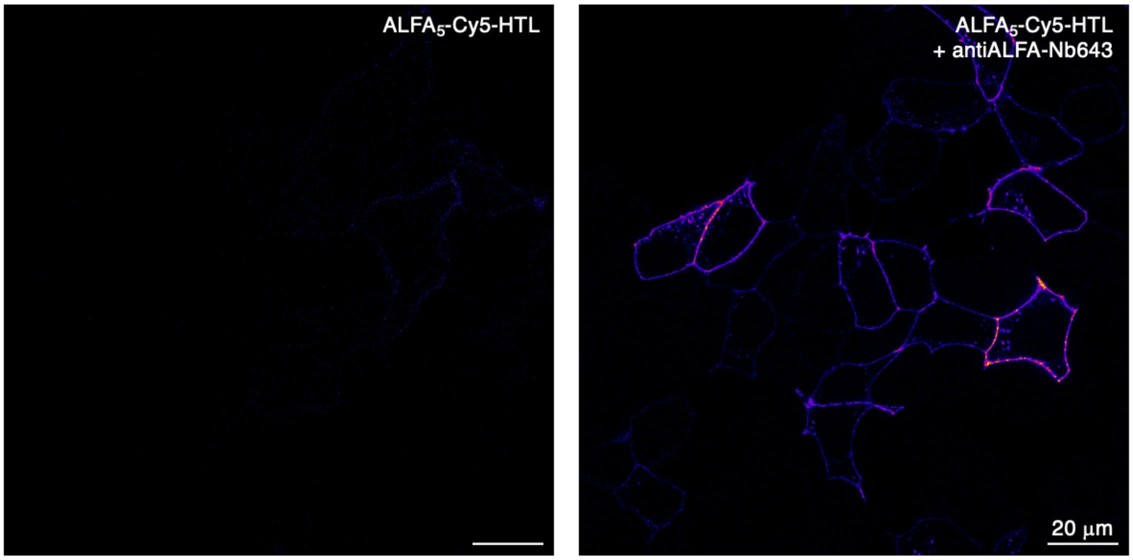

Supplemental Figure 1. Confocal images of SNAP-HTP-mGluR2 transfected HEK293 cells on a Leica SP8 TCS STED FALCON.

6. Cell culture and imaging

HEK293T cells (70,000) were seeded on 8-well, PLL coated ibidi dishes in full media (DMEM high glucose, stable glutamax, 10% FCS) and transfected the next day with 50 ng of plasmid SNAP-HTP-mGluR2,[2] using JETPrime (VWR) according to the manufacturer's instructions. Medium was exchanged after 4 hours post transfection, and cells were incubated overnight at 37 °C. The next day, cells were stained with 1 μM SBG-OG[3] and500 nM $ALFA_5$-Cy5-HTL and 1 μM Hoechst33342, or 1 μM AF488-HTL and $ALFA_5$-Cy5-BG and 1 μM Hoechst33342 for 30 min at 37 °C, before washing once with media. Cells were fixed using 2% PFA (Alfa Aesar) for 20 min at room temperature and washed 3 times with PBS afterwards. The incubation with the anti-ALFA-Nb643 (Nano-Tag, Fluo-Tag-X2 anti-Alfa, ATTO643) was performed over night at 0 °C. Cells were washed 3 times with PBS and fixed again with 2% PFA for 20 minutes. Cells were washed again 3 times with PBS and imaged in PBS on a Nikon CSU-X1 using a 40x objective (air), equipped with a EMCCD camera (Andor AU-888), fast triggered acquisition and a piezo Z-drive for fast imaging. Incubator (OKOLAB) was set to 37 °C with 5% CO_2. Lasers used: 405 nm 488 nm, 561 nm, and 638 nm. STED was performed on a Leica SP8 TCS STED FALCON (Leica Microsystems) equipped with a pulsed white-light excitation laser (80 MHz repetition rate, NKT Photonics), a 100 objective (HC PL APO CS2 100/ 1.40 NA oil), operated by LAS X. λ_{Ex} = 640 nm λ_{Em} = 655–748 nm. The confocal images were collected using a time gated Hybrid detector (0.5–6 ns).

7. Non-denaturating SDS-PAGE

Peptides ALFA5-Cy5-HTL or ALFA5-Cy5-BG were diluted to 5 nM in PBS and incubated with anti-ALFA-Nb643 (0, 0.1, 1, 10 or 100 nM) for 30 min at room temperature. The samples were diluted with a non-reducing loading buffer (Carl Roth) and loaded onto a gradient gel (4–20% Mini-PROTEAN® TGX™ Precast Protein Gel) without boiling. The anti-Alfa Nb643 was also loaded in a concentration of 100 nM. A low range marker (Thermo Fisher) was applied to estimate the size of the peptides. The gel was imaged at a fluorescence scanner using 635 nm and 400V laserpower.

8. Image Calculation and Statistics

For image representation FIJI was used with the integrated *Calculator Plus* to obtain ratiometric images. Statistics were performed in Prism 10.

9. Excitation and Emission profiles

ALFA$_5$-Cy5-HTL or ALFA$_5$-Cy5-BG were dissolved in DMSO to a concentration of 1 mM and diluted in PBS (pH = 7.4) to obtain a concentration of 200 nM. 200 µL solution was transferred into Greiner black flat bottom 96 well plate and excitation and emission profiles were recorded on a TECAN INFINITE M PLEX plate reader (λ_{Ex} = 605±10 nm; λ_{Em} = 640–800±20 nm; 10 flashes; 20 µs integration time). Data normalization, integration and plotting was performed in GraphPad Prism 8.

ALFA$_5$-Cy5-HTL: $\lambda_{max\ Ex}$ = 651 nm; $\lambda_{max\ Em}$ = 674 nm

ALFA$_5$-Cy5-BG: $\lambda_{max\ Ex}$ = 650 nm; $\lambda_{max\ Em}$ = 668 nm

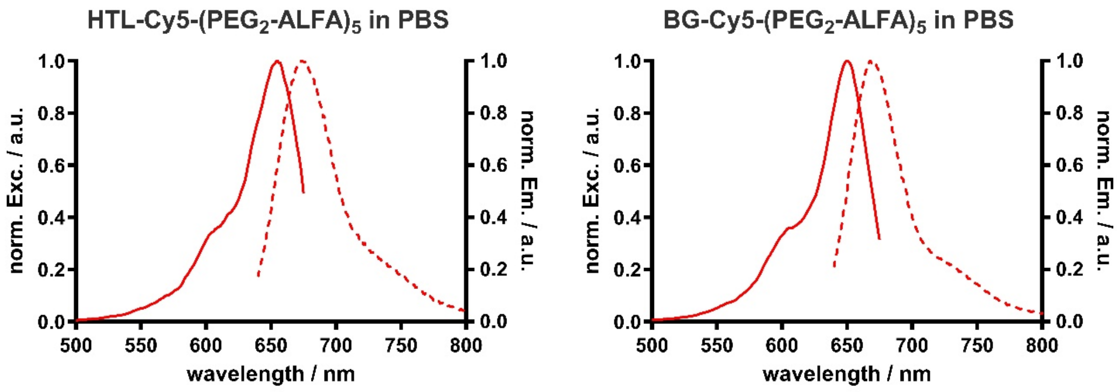

10. References

[1]R. Birke, J. Ast, D. A. Roosen, J. Lee, K. Roßmann, C. Huhn, B. Mathes, M. Lisurek, D. Bushiri, H. Sun, B. Jones, M. Lehmann, J. Levitz, V. Haucke, D. J. Hodson, J. Broichhagen, *Org. Biomol. Chem.* **2022**, *20*, 5967–5980.

[2]K. Roßmann, R. Birke, J. Levitz, B. Jones, J. Broichhagen, *RSC Chem. Biol.* **2025**, 10.1039.D4CB00209A.

[3]P. Poc, V. A. Gutzeit, J. Ast, J. Lee, B. J. Jones, E. D'Este, B. Mathes, M. Lehmann, D. J. Hodson, J. Levitz, J. Broichhagen, *Chem. Sci.* **2020**, *11*, 7871–7883.
